# What the Papers Say

**DOI:** 10.1093/jhps/hnw020

**Published:** 2016-06-13

**Authors:** Ajay Malviya

The Journal of Hip Preservation Surgery (JHPS) is not the only place where work in the field of hip preservation may be published. Although our aim is to offer the best of the best, we continue to be fascinated by work that finds it way into journals other than our own. There is much to learn from it so *JHPS* has selected six recent and topical articles for those who seek a brief summary of what is taking place in our ever-fascinating world of hip preservation. What you see here are the mildly edited abstracts of the original articles, to give them what *JHPS* hopes is a more readable feel. If you are pushed for time, what follows should take you no more than 10 minutes to read. So here goes …

## IS THE TONNIS CLASSIFICATION USEFUL IN HIP PRESERVATION SURGERY?

The Tönnis classification is widely accepted for grading hip arthritis, but its usefulness as a reference in hip-preserving surgery is yet to be demonstrated. Valera *et al* [1] from Barcelona, Spain aimed to evaluate the reproducibility and reliability of the Tönnis classification in early stages of hip osteoarthritis.

Three orthopaedic surgeons with different levels of experience examined 117 hip X-rays that were a random mix of two groups: a group of 31 candidates for hip-preserving surgery and a control group of 30 patients that were asymptomatic with respect to the hip joint. The surgeons were asked to rate the degree of osteoarthritis according to the Tönnis classification. After 2 months, the surgeons were asked to re-evaluate the X-rays in a random order. Intra- and interobserver reliabilities were calculated by comparing the observers' two estimations using Kappa statistics.

Kappa values for interobserver reliability were slight or fair (range 0.173–0.397). Kappa values for intraobserver reproducibility were fair (range 0.364–0.397). Variance in grading no and slight osteoarthritis was the most frequent cause for intra- and interobserver disagreements (76.3 and 73.01% of the non-concordant observations, respectively). The confidence interval analysis revealed that the observers' experience did not affect reproducibility.

The authors concluded that the Tönnis classification is a poor method to assess early stages of hip osteoarthritis and suggested that its routine use in therapeutic decision-making for conservative hip surgery should be reconsidered.

## HIP PRESERVATION SURGERY FOR AVASCULAR NECROSIS OF THE FEMORAL HEAD—THE ROLE OF AUTOLOGOUS BONE MARROW AND ULTRASOUND TREATMENT

Japanese researchers [2] have reported on the safety and efficacy of a novel combined autologous concentrated bone marrow grafting and low-intensity pulsed ultrasound (LIPUS) in the treatment of osteonecrosis of the femoral head (ONFH).

The study includes seven males and seven females (mean age: 40 years; 22 hips) with ONFH, who underwent autologous concentrated bone marrow grafting followed by 6 months of continuous LIPUS. The mean follow-up period was 26 months. The authors evaluated site-specific bacterial infection of the grafted bone marrow concentrate microbiologically and site-specific cancer by magnetic resonance imaging 24 months after grafting. All patients were assessed using the visual analogue scale (VAS) for pain and the Japanese Orthopedic Association (JOA) hip score. Clinical and plain radiographic evaluations were performed before grafting and at the most recent follow-up. Computed tomography (CT) scans were obtained before and 12 months after grafting.

None of the grafted bone marrow concentrates were infected, and none of the patients developed a tumor at the treatment site. The VAS and JOA scores improved in all patients. Collapse progressed in eight of the 22 hips, but none required total hip arthroplasty. The mean volume of new bone formation 12 months post-grafting as seen on CT was 1256 mm^3^. New bone formation was observed in all patients.

The authors concluded that this technique was safe and effective as a joint-preservation option for patients with ONFH.

## WHAT IS THE EXPECTED OUTCOME AFTER ARTHROSCOPIC TREATMENT OF FEMOROACETABULAR IMPINGEMENT IN PATIENTS WITH BORDERLINE HIP DYSPLASIA?

Nawabi *et al.* [3] from the Center for Hip Preservation at Hospital for Special Surgery, New York, compared the outcomes after hip arthroscopy for femoroacetabular impingement (FAI) in borderline dysplastic (BD) patients with a control group of nondysplastic patients.

Forty-six patients (55 hips) with BD (Lateral centre-edge angle [LCEA], 18°–25°) were included and compared with an age- and sex-matched control group (LCEA, 25°–40°) of 131 patients (152 hips). Patient-reported outcome scores, including the modified Harris Hip Score (mHHS), the Hip Outcome Score-Activities of Daily Living (HOS-ADL) and Sport-Specific Subscale (HOS-SSS), and the International Hip Outcome Tool (iHOT-33), were collected preoperatively and at 1 and 2 years postoperatively.

The mean LCEA was 22.4° ± 2.0° (range, 18.4°–24.9°) in the BD group and 31.0° ± 3.1° (range, 25.4°–38.7°) in the control group (*P* < 0.001). The mean preoperative alpha angle was 66.3° ± 9.9° in the BD group and 61.7° ± 13.0° in the control group (*P* = 0.151). Cam decompression was performed in 98.2% and 99.3% of cases in the BD and control groups, respectively; labral repair was performed in 69.1% and 75.3% of the BD and control groups, respectively, with 100% of patients having a complete capsular closure performed in both groups. At a mean follow-up of 31.3 ± 7.6 months (range, 23.1–67.3 months) in unrevised patients and 21.6 ± 13.3 months (range, 4.7–40.6 months) in revised patients, there was significant improvement (*P* < 0.001) in all patient-reported outcome scores in both groups. Multiple regression analysis did not identify any significant differences between groups. Importantly, female sex did not appear to be a predictor for inferior outcomes. Two patients (4.3%) in the BD group and six patients (4.6%) in the control group required revision arthroscopy during the study period.

The paper concluded that favorable outcomes can be expected after the treatment of impingement in patients with borderline dysplasia when labral refixation and capsular closure are performed, with comparable outcomes to nondysplastic patients. However, they did identify the need for further follow-up in larger cohorts to prove the durability and safety of hip arthroscopy in this challenging group and to further explore potential sex-related differences in outcome.

## WHAT DOES THE LITERATURE SAY ABOUT PATIENT POSITIONING DURING HIP ARTHROSCOPY?

A multicentred collaboration between units in Canada, US and Sweden has performed a systematic review [4] comparing supine and lateral decubitus positions for hip arthroscopy. This review examined outcomes and risk profiles of hip arthroscopy in supine versus lateral decubitus positions to elucidate any superiority of one approach over the other.

Three databases (Embase, PubMed and Medline) were searched for studies that addressed hip arthroscopy performed in either position, and were subsequently screened by two reviewers.

Similar outcomes were observed. Supine studies showed a greater mean postoperative improvement for modified Harris hip score (33.74), visual analog scale (-3.99), nonarthritic hip score (29.61), Harris hip score (35.73) and hip outcome score (31.4). Lateral decubitus studies showed greater improvement using the Western Ontario and McMaster University Osteoarthritis (14.76) score. Supine studies reported more neuropraxic injuries (2.06% vs. 0.47%), labral penetration (0.65% vs. 0%) and heterotopic ossification (0.21% vs. 0%). Lateral decubitus studies reported more fluid extravasation (0.21% vs. 0.05%) and missed loose bodies (0.08% vs. 0.01%). Similar rates of revision (1.8% lateral and 1.4% supine) and conversion to open procedures (2.6% in lateral and 2.0% in supine) were also identified.

The authors concluded that because of the quality of evidence, direct comparisons were limited and, therefore, literature did not establish the superiority of one approach over the other. However, the supine position was associated with more neuropraxic injuries, labral penetration and heterotopic ossification, whereas lateral decubitus has increased risk of fluid extravasation and missed loose bodies.

## HOW DOES INTRAOPERATIVE FLUOROSCOPY COMPARE WITH STANDARD RADIOGRAPHY TO EVALUATE ACETABULAR MORPHOLOGY DURING SURGERY?

Swiss and American researchers [5] have attempted to compare quantitative measurements of acetabular morphology obtained using intraoperative fluoroscopy, to standardised anteroposterior (AP) pelvis radiographs in cadaveric specimens.

Ten dried human pelvis specimens (20 hips) were imaged using hip-centered fluoroscopy and standardised AP pelvis radiographs. Each hip was evaluated for acetabular version and coverage, including lateral center edge (LCE) angle, acetabular index (AI), total anterior and posterior coverage, and crossover sign.

No statistically significant differences existed between the mean LCE angle (fluoroscopy 36.5° vs. plain films 36.1°, *P* = 0.59), AI (0.6° vs. 0.2°, *P* = 0 .61), ACM (acetabular depth measurement) angle (44.0° vs. 44.1°, *P* = 0.89), Sharp's angle (31.8° vs. 32.4°, *P* = 0.44) and the total femoral coverage (80.9% vs. 80.7%, *P* = 0.83). Conversely, total anterior coverage (30.7% vs. 33.3%, *P* < 0.0001) appeared significantly decreased and the total posterior coverage (54.1% vs. 49.1%, *P* < 0.0001) appeared significantly increased in fluoroscopy compared with plain film radiographs. Fluoroscopy also failed to identify the presence of a crossover sign in 30% and underestimated the retroversion index (9% vs. 13%, *P* = 0.016).

The authors concluded that fluoroscopy is reliable for measurement of LCE angle and AI; however, fluoroscopy leads to a more anteverted projection of the acetabulum with significantly decreased total anterior coverage, significantly increased total posterior coverage, and underestimated signs of retroversion compared with standardised AP pelvis radiography. Surgeons should be mindful of this during hip preservation surgery.

## BERNESE PERIACETABULAR OSTEOTOMY—DOES SURGICAL APPROACH INFLUENCE OUTCOME?

Luo *et al.* [6] from Beijing, China have compared three main approaches of Bernese periacetabular osteotomy: improved ilioinguinal (I-I) approach, two-incision Smith–Petersen (TSP) approach and modified Smith–Petersen (MSP) approach.

In this retrospective case control study, 101 hips (95 patients) had Bernese periacetabular osteotomy performed using these three approaches. The outcome measures used were the operative time, blood loss, intraoperative and postoperative allogeneic blood transfusion and postoperative complications (infection, sciatic nerve irritation, lateral cutaneous nerve injury, reoperation, screw failure and posterior column fracture).

The authors report that among the three approaches, I-I had less operation time and more blood loss (*P* < 0.05), TSP had less blood loss (*P* < 0.05) but more complications, and MSP had less blood loss (*P* < 0.05) and less complications (sciatic and lateral cutaneous nerve injury). There was no statistical difference between the patient demographics and radiological correction achieved.

They concluded that the MSP approach is superior to the other two approaches in doing periacetabular osteotomy.
